# Empowerment in primary care and psychiatric settings: a psychometric evaluation of the Swedish version of the empowerment scale

**DOI:** 10.1186/s40359-025-03123-y

**Published:** 2025-08-13

**Authors:** Linnea Nissling, Magnus Lindwall, Viktor Kaldo, Pernilla Larsman, Lars Hansson, Sandra Frööjd, Marie Bendix, Sandra Weineland

**Affiliations:** 1 Department of Psychology, Faculty of Social Sciences, University of Gothenburg, Gothenburg, Sweden; 2 Research, Development, Education and Innovation, Primary Health Care, Region Västra Götaland, Sweden; 3 General Practice/Family Medicine, School of Public Health and Community Medicine, Institute of Medicine, Sahlgrenska Academy, University of Gothenburg, Gothenburg, Sweden; 4 The Swedish School of Sport and Health Sciences (GIH), Stockholm, Sweden; 5 Centre for Psychiatry Research, Department of Clinical Neuroscience, Karolinska Institutet, & Stockholm Health Care Services, Region Stockholm, Stockholm, Sweden; 6 Department of Psychology, Faculty of Health and Life Sciences, Linnaeus University, Växjö, Sweden; 7 Department of Health Sciences, Lund University, Lund, Sweden; 8 Department of Clinical Sciences/Psychiatry, Umeå University, Umeå, Sweden

**Keywords:** Patient empowerment, Person-centered care, Measurement instruments, Primary care, Psychiatric care

## Abstract

**Background:**

There has recently been an increased emphasis on patient empowerment and collaboration within their healthcare. However, there is widely a lack of clarity to the concept of empowerment and existing measurement tools lack uniformity, covering diverse domains and related concepts.

**Objectives:**

This study aims to conduct a psychometric evaluation of the Swedish version of the Empowerment Scale– Making Decisions, focusing on its structural validity and reliability in assessing patient empowerment. This includes a detailed examination of the factor structure across two different contexts, psychiatric care (*n* = 211) and primary care (*n* = 210). We will compare several confirmatory factor analysis (CFA) models proposed in previous research to identify the best fit. If no models provide a good fit, we intend to suggest a new scale for further evaluation.

**Method:**

The dimensionality of the scale was tested by comparing four CFA models, together with a one-factor solution, to identify the best fit for the two samples. Reliability measures were determined by coefficient Omega (ω) as well as Cronbach’s alpha (α).

**Results:**

There was limited support for the one-factor solution in both samples, challenging the scale’s assumed unidimensionality (p*rimary care sample*: x^2^(350) = 1074, *p* <.001, CFI = 0.58, TLI = 0.54, RMSEA = 0.10 (90% CI: 0.09 − 0.11), SRMR = 0.11; *psychiatric care sample*: (x^2^(350) = 1307, *p* = < 0.001, CFI = 0.66, TLI = 0.63, RMSEA = 0.11 (90% CI:0.11;0.12), SRMR = 0.10). None of the previously suggested factor solutions demonstrated satisfactory fit. However, a three factor-solution entailed the less complexity and best model fit (p*rimary care sample*: (x^2^(270) = 503, *p* = < 0.001),CFI = 0.85, TLI = 0.84, RMSEA = 0.06 (90% CI 0.06;0.07), SRMR = 0.07; *psychiatric care sample*: (x^2^(270) = 622, *p* <.001), CFI = 0.87, TLI = 0.86, RMSEA = 0.08 (90% CI 0.07;0.09), SRMR = 0.07). Based on this, we continued with exploratory refinements of this solution and arrived at two adjusted three-factor models based on each sample. These two adjusted models displayed only slight differences, and in a last step we removed the items that differed between the samples to arrive at one solution appropriate for use in health care settings in general. As a result, an improved and shortened adaptation of the scale was put forward that included 18 items targeting the subscales *Self-Esteem*, *Powerlessness* and *Activism*. This solution remained relatively clear to the previously proposed solutions (*primary care sample*:(x^2^(131) = 240, *p* <.001), CFI = 0.91, TLI = 0.90, RMSEA = 0.06 (90% CI 0.05;0.08), SRMR = 0.07; *psychiatric care sample*: (x^2^(131) = 379, *p* <.001), CFI = 0.88, TLI = 0.86, RMSEA = 0.09 (90% CI 0.08;0.10), SRMR = 0.07; *combined sample*: (x^2^(131) = 432, *p* <.001), CFI = 0.91, TLI = 0.90, RMSEA = 0.07 (90% CI 0.07;0.08), SRMR = 0.06).

**Conclusion:**

The results reinforce the difficulties in measuring empowerment given the complexity of this concept. The improved and shortened adaptation of the scale could potentially be used within health care settings to measure empowerment, but further research is needed to conceptualize and measure empowerment in patients with mental health problems. Given scarce support for the scale’s unidimensionallity, future research should explore using multiple instruments targeting different constructs to measure patient empowerment more comprehensively.

**Supplementary Information:**

The online version contains supplementary material available at 10.1186/s40359-025-03123-y.

Person-centered care is a tenet within today’s health care. A person-centered care involves patients, their families, and healthcare professionals collaborating to provide individually tailored and coordinating care based on the patient’s needs, circumstances, and preferences. It aims to help patients recognize their strengths and abilities for independent living [1]. Patient empowerment can be understood as the patient’s ability to take control over the management of their disease and their ability to participate and be involved in decisions regarding their care and treatments based on knowledge, skills and self-awareness [2]. The notion of patient empowerment is however not limited to the individual micro level but also entails patient participation and involvement at the meso level, within organizations and hospitals for example, and at the macro level by collectively taking political, social and cultural action, for example in policymaking or healthcare research [2].

Despite the emphasis on empowerment within health care settings, there is widely a lack of clarity to the concept [3]. It entails a complexity since it is seen as a concept built up by several dimensions, and additionally entailing both an individual and a collective level [4]. The empowerment-concept has been used and researched across a range of fields, including psychology, social science, political science, health promotion and economics, consequently resulting in different use and meaning [5]. It has psychological, social, and political dimensions. The political dimension of empowerment refers to the ways in which individuals or groups gain control over decisions and actions that affect their lives, particularly in relation to power structures and institutional governance [6]. In the context of healthcare, this dimension involves patients having a voice in decision-making processes that influence their care, advocating for their rights, and participating in shaping healthcare policies. It is about shifting the balance of power from healthcare providers or systems to the patients themselves, enabling them to act as agents of change within the system. Empowerment can be seen as a state/outcome, the extent to which a person or group feels empowered, or as a process, the effect of certain factors or events on empowerment and consequently its significance for achievement of another goal [4]. An empowered patient (i.e., state/outcome) feels confident enough to participate in their own health care decisions or self-management of their illness (although this does not necessarily mean that the patient actually participates). Empowerment as a process means that the patient is becoming enhanced in their capacity to critically evaluate and make informed decisions [7].

Bravo and colleagues [8] developed a conceptual map of patient empowerment and its relation to related and partly interacting concepts based on previous litterature and interviews with stakeholders such as patient representatives, primary care clinicians, health managers and health service researchers. They found that empowerment on one hand entails ethical assumptions about patients’ rights, opportunities and responsibilities to autonomy, self-determination, and an equal power-balance within the patient-clinician relationship. Empowering processes for enhancing patients’ sense of empowerment include interventions for shared decision making, motivational interviewing, coaching or education directed towards self-management of the disease. Indicators that patients are/have become empowered include capacities or states such as self-efficacy, knowledge, health literacy, feeling respected and a sense of meaning and coherence in relation to their disease, or actual behaviors like participating in shared-decision making, self-managing their own health or taking active control over their situation by for example researching information about their disease or participating in support groups. Lastly, outcomes of empowerment could be immediate, intermediate and long-term. Behaviours like shared decision-making and active participation in own care could be considered immediate outcomes of empowerment. Patient outcomes, like wellbeing or quality of life, could be considered intermediate outcomes, and health status as long term outcomes of becoming more empowered [8].

Indeed, as there is little consensus on how the concept should be defined and understood, there are also few universal measures of empowerment that can be used to evaluate interventions to increase empowerment in various health care settings [9]. A systematic literature review of empowerment measurements showed that the domains that were covered ranged from one to eleven domains, and included aspects such as control, competence, participation, power, motivation, political efficacy, leadership, and positive relationships, among others [5]. A systematic review of existing measurement scales for patient empowerment found that the 19 measurement scales included together encompassed 38 different underlying concepts, calling for research to develop a more coherent definition of patient empowerment [9]. In yet a recent systematic review the broad range of definitions and measurement scales identified underscored the lack of consensus of a definition of patient empowerment as well as methodological limitations in studies examining predictive correlates of empowerment as well as its association with treatment outcomes [10]. Consequently, some researchers in the field argue that empowerment must be understood based on the context in which it is operationalized, and that the concept can have different meanings in different groups, people and situations. They empasize that it is neither possible nor desirable to develop general measurement instruments to capture empowerment, but that measuring instruments need to be specifically adapted to the context in which empowerment is intended to be measured [4].

The Empowerment Scale - “Making Decisions Scale” was originally developed by Rogers and colleagues [11] in an American context of psychiatric care and intended to measure personal empowerment for patients with mental illness. The scale is commonly used as one of few existing scales of empowerment within the mental health field. The scale and corresponding items were developed in collaboration with a reference group of leaders from different consumer-survivor movements. The original 28-item scale generally shows high internal consistency with Cronbach’s Alpha ranging from α = 0.82-0.86 [11, 12, 13, 14, 15] and good construct validity with positive associations with measures including quality of life, social support and self-esteem, and negative associations with needs for care and psychiatric symptoms [11, 12, 13, 14, 15, 16, 17, 18]. However, less consistent Cronbach’s alpha reliability has been reported for the subscales across studies, with Cronbach’s alpha ranging from 0.45 to 0.91 for the different subscales [12, 13, 14, 15]. In particular, Cronbach’s alpha for the subscale power-powerlessness has been found to be weak in several studies [13, 14, 15]. Furthermore, studies examining the factor structure of the scale have identified several different solutions, leading to various proposed adaptations of the scale with differing items. These factor solutions are described in detail in the methods section of this study. This is further complicated by the fact that the scale has been assessed across various contexts and populations, including different countries, diverse backgrounds, distinct theoretical perspectives of researchers, and varying statistical analysis methods (e.g., principal component analysis vs. CFA, orthogonal vs. oblique rotation).

The scale has been validated in people with severe mental illness, such as patients from consumer-operated programs in the U.S with serious mental illness of schizophrenia, schizoaffective disorder or major depression [12], an U.S hospitalization program with participants with serious and persistent mental illness [16], psychiatric inpatients or outpatients in the Netherlands with a psychotic disorder [13], a sample of Portuguese patients mainly diagnosed with schizophrenia, bipolar disorder or depression using psychosocial or community support programs [17] and a sample of psychiatric outpatients in the U.S [15]. A Swedish version of the scale was developed by Hansson and Björkman [14] and has been validated in a sample of psychiatric patients with severe mental illness. In that study 60% of the participants had a diagnosis of Schizophrenia and an additional 20% presented with other psychosis diagnoses [14]. Thus, the scale has previously mainly been evaluated in psychiatric populations for people with severe mental illness, including the Swedish version of the scale [14].

The Empowerment Scale– “Making Decisions Scale” is designed to assess how patients perceive their ability to participate in decision-making processes. This is a key element of empowerment and is particularly relevant in healthcare environments, where patients’ involvement in their own treatment plans and person-centered care is increasingly prioritized. While other measures of quality of life, well-being, and self-esteem exist, the specificity of this scale is particularly important for understanding how healthcare settings and interventions impact patients’ capacity to make informed, autonomous decisions about their care. However, since the perception of empowerment may be strongly influenced by the context and the population considered, it is crucial to assess whether the scale accurately measures empowerment across various healthcare settings.

Consequently, our goal was to investigate if the Empowerment scale - “Making decisions scale” could be used as a broad measurement for empowerment within health care settings for patients with mental health issues. Psychiatric and primary care patient populations exhibit notable distinctions in several aspects. Psychiatric populations predominantly comprise individuals with severe and chronic mental health conditions, often requiring specialized treatment and care. In contrast, primary care populations consist of patients with a broader spectrum of medical conditions, including those of varying severity. They primarily receive healthcare services in general medical settings, such as family medicine clinics or community health centers. In psychiatric care, the emphasis often lies on symptom management, medication, and psychotherapeutic interventions, while primary care focuses on holistic health, preventive measures, and addressing a wide range of medical concerns. However, despite differences, the fundamental objective in both settings is to provide patient-centered care that respects individual autonomy and preferences, and the concept of empowerment remains relevant.

## Objectives

The aim of this study is to conduct a psychometric evaluation of the Swedish adaptation of the Empowerment Scale– Making Decisions, focusing on its structural validity and reliability in assessing patient empowerment in health care settings. Specifically, we aim to compare the factor structure and internal consistency across two distinct populations among psychiatric and primary care patients. We seek to compare multiple confirmatory factor analysis (CFA) models to identify the best fit for the sample. If no models provide a good fit, we intend to suggest a new scale for further evaluation.

## Method

### Measurements

Empowerment was measured by *the Empowerment Scale - Making Decisions* [11]. It is a self-assessment scale constructed to measure personal empowerment among users of mental health services. The scale consists of 28 items answered on a 4-point Likert scale, from strongly agree = 1 to strongly disagree = 4. Sum scores range from 28 to 112, with higher scores indicating a stronger sense of empowerment. An overall empowerment score is calculated by either summing all item-scores or by summing them and dividing them by the total number of items to arrive at an average item-score. The original scale consists of five subscales derived from a principal component analysis by Rogers and colleagues [11]: Self-Esteem-self-efficacy (nine items), Power-powerlessness (eight items), Community activism and autonomy (six items), Optimism and control over the Future (four items), and Righteous anger (four items). Examples of items include “I am usually confident about decisions I make” (Self-Esteem-self-efficacy), “Experts are in the best position to decide what people should do or learn” (Power-powerlessness), “People working together can have an effect on their community” (Community activism and autonomy), “People are limited only by what they think possible” (Optimism and control over the future) and “Getting angry about something never helps” (Righteous anger). A list of all 28 items is found in Appendix I. Cronbach’s alpha in the original study was α = 0.86. for the whole scale [11].

### Participants

The Empowerment scale - Making decisions [11] was evaluated using self-ratings in two samples of participants recruited for different studies conducted within Swedish primary care and psychiatric care. For both samples, the empowerment-ratings were collected before the participants took part in any intervention. Table 1 shows demographic variables of the participants.

**Table 1:** Demographic variables of participants

Variable		Primary care (sample 1) (*n =* 210)	Psychiatry (sample 2) (*n* = 221)
**Age**			
Frequencies (%)	18–25 years	25 (11.9)	15 (6.8)
	26–35 years	122 (58.1)	56 (25.3)
	36–45 years	47 (22.4)	72 (32.6)
	46–55 years	10 (4.8)	49 (22.2)
	56–65 years	3 (1.4)	24 (10.9)
	66–75 years	2 (0.9)	2 (0.9)
	Older than 75 years	-	2 (0.9)
	Missing	1 (0.5)	1 (0.4)
**Gender**			
Frequencies (%)	Man	24 (11.4)	122 (55.2)
	Woman	186 (88.6)	99 (44.8)
**Employment status**			
Frequencies (%)	Working	164 (78.1)	30 (13.6)
	Student	13 (6.2)	18 (8.1)
	Parental leave	5 (2.4)	-
	Unemployed/sick leave/retirement/household activities	28 (13.3)	163 (73.8)
	Other	-	8 (3.6)
	Missing	-	2 (0.9)

### *The primary care sample* (sample 1; *n* = 210)

The primary care sample consisted of participants assembled from four different subsamples. A first sample (*n* = 55) comprised participants recruited from primary care patients with anxiety disorders for a randomized controlled study assessing an internet-delivered CBT (ICBT) intervention that was tailored based on participants’ self-selection of treatment structure, content and contact with their clinician [19]. Another sample (*n* = 9) included participants recruited for an intervention study on the addition of peer support workers in internet-delivered CBT (ICBT) treatment for anxiety disorders [20]. A third sample (*n* = 37) of participants was recruited specifically for the current study while seeking initial mental health assistance at their primary care center between 2021 and 2022. A fourth sample (*n* = 109) comprised women who registered an interest in an, to this date ongoing, study on internet-delivered CBT (ICBT) for pregnant women with depression. The purpose of that study is to evaluate whether different person-centered adaptations can increase treatment effects and decrease risk for postpartum depression. The participants for that study were recruited through advertisements on internet, in media, at maternity care centers, in a mobile application for pregnant women and through referments from primary health care centers and psychiatric outpatient clinics. The patient group is largely representative of a primary care population, but the treatment itself is carried out at a clinic for internet psychiatry in Sweden.

### *The psychiatric sample (sample 2*; *n* = 221)

The psychiatric sample consisted of participants from four different subsamples of patients in contact with Swedish mental health services. A first sample (*n* = 40) was included in a feasibility study performed as a prequel to a later randomized controlled trial (RCT). This RCT was performed as an EU study testing patient involvement in treatment where the Swedish sample included *n* = 59 participants (subsample 2). Both these samples included patients diagnosed with schizophrenia [21]. The third subsample (*n* = 70) consisted of patients included in a study evaluating effects of a mental health rehabilitation model [22]. The last subsample (*n* = 52) emanated from a RCT evaluating the effectiveness of a case management model [23].

### Data analysis

The data program *JASP* was used for statistical analyses.

### Internal consistency

Reliability measures were determined by coefficient Omega (ω) as well as Cronbach’s alpha (α). Omega has been proposed as a better measure of internal consistency [24] and is thus presented along with the more well-known Cronbach’s alpha.

### Confirmatory factor analysis

The dimensionality of the scale was tested by using confirmatory factor analysis (CFA). Based on previous research, we identified four different factor models that have been proposed for the empowerment scale and could be subjected to CFA, see further details below. We tested these models in each of the two samples (primary care and psychiatric care). In addition to testing *model 1–4*, we added a one-factor solution applied to the original 28 items in the two samples, denoted as “*model 0*” henceforth. This choice was motivated by the consideration that, in practice, a singular total score is computed for the entire instrument, which assumes that there is only one underlying factor. However, a one-factor solution has to our knowledge not previously been tested.

### Model 1– five factor solution based on 28 items [11, 14, 15]

Rogers and colleagues [11] originally found a five-factor solution through a factor analysis using principal component analysis and oblique rotation, which included the factors of *Self-Esteem-Self-efficacy*, *Power-powerlessness*, *Optimism and control over the future*, *Community activism* and *Righteous anger*. In the model by Roger and colleagues [11], item 7 and 10 cross-loaded on both the Self-esteem–self-efficacy and Righteous anger factor, and item 27 cross-loaded on both the Community activism and autonomy and Optimism and control over the future factors. Internal consistency for the whole scale was α = 0.86 in the original study by Rogers and colleagues [11]. The five-factor solution has also been validated in the Swedish version of the Scale by Hansson & Björkman [14]. They also used principal component factor analysis with varimax rotation and the same cross-loadings and found a five-factor solution with two superordinate factors, one revolving around “self-esteem and activism” (α = 0.89) and one directed towards “community and power” (α = 0.64 [14]),. Internal consistency in that study was α = 0.84 for the total scale, with varying results for the subscales (Self-esteem–self-efficacy: α = 0.90; Activism and autonomy: α = 0.68; Optimism and control over future: α = 0.64; Righteous anger: α = 0.64; Power–powerlessness: α = 0.45 [14]).

### Model 2– A five-factor solution based on 25 items [12]

In a later reliability study by 2010, Rogers and colleagues [12] first used a confirmatory factor analysis to test the five-factor solution. Since the CFA did not provide acceptable fit, they continued with an exploratory factor analysis with principal-components analysis and varimax (orthogonal) rotation and removed two items (2 and 15) based on low factor loadings and a third item (21) because it was deemed to not resonate well in other cultures outside of the U.S. They then performed a last confirmatory factor analysis for this shorter 25-items version on another randomly selected half of the original sample with the same cross-loadings as in model 1. The fit statistics showed a mediocre fit with the five-factor structure (goodness-of-fit = 0.88, CFI = 0.84, RMSEA = 0.07, NNFI = 0.81), but it was considered the best solution compared to a four- or three factor solution which had also been tested [12]. Internal consistency was α = 0.82 for the total scale and ranging from fair to excellent for the subscales (self-esteem: α = 0.82; power and powerlessness: α = 0.59; community activism and autonomy: α = 0.59; optimism and control over the future: α = 0.45; righteous anger: α = 0.64; [12]).

### Model 3– A five-factor solution based on 20 items [17]

Jorge-Monteiro and colleagues [17] validated the shortened 25-item version proposed by [12] and made further adjustments based on the results from the confirmatory factor analysis. They removed an additional 5 items from the 25-item version (2, 7, 15, 20, 23) based on low factor loadings. The adjusted 20-item version showed reasonable fit statistics (χ2 = 1.78; CFI = 0.90; TLI = 0.88; GFI = 0.89; PCFI = 0.75; RMSEA = 0.06, 90% CI [0.05; 0.07]). Internal consistency for the scale was (α = 0.79) and ranged from fair to good for subscales (self-esteem: α = 0.87; power: α = 0.56; activism: α = 0.72; optimism: α = 0.52; anger: α = 0.55 [17]).

### Model 4– A three-factor solution based on 25 items [18]

Morris and colleagues [18] tested the scale and measurement equivalence between white and black participants in an American sample of outpatients with severe mental illness. They first used a confirmatory factor analysis on half of their sample (the white participants) based on the original 28-item version of the scale to test the five-factor solution proposed by [11]. Because this CFA did not provide acceptable fit, they continued with an explorative factor analysis with oblique (oblimin) rotation on a randomly selected half of the white sample and arrived at a three-factor solution comprising the subscales *Self-esteem* (α = 0.88), *Powerlessness* (α = 0.68) and *Activism* (α = 0.71). Based on the modification indices on a subsequent confirmatory factor analysis on the other half of the white sample, three items (2, 15, 23) were removed because of low factor loadings. Additionally, a residual correlation was allowed between items 16 and 22 and item 26 was allowed to cross load on both the Self-esteem and Activism factors [18]. The fit statistics were reported to be mixed (*χ*^2^_(540) =_ 1533; CFI = 0.86; RMSEA = 0.05; [18]).

### Model testing

The maximum likelihood estimator was used for model testing. The chi-square goodness-of-fit statistic test was used for estimating the overall fit of the model. However, as this goodness-of-fit measure tends to be over-sensitive to sample size [25], we also used alternative goodness-of-fit statistics. This included the comparative fit index (CFI; [26]), the Tucker-Lewis index (TLI [27],, the root mean square error of approximation (RMSEA; [28] and the 90% confidence intervals of the RMSEA, and the standardized root mean square of the residuals (SRMR; [26]).

For CFI and TLI, values close to 0.95 have been proposed as cut-off for good model fit [29], although values above 0.90 are considered acceptable [26]. For the root mean square error of approximation (RMSEA) lower values are desirable, with values below 0.06 (including also the upper limit of the 90% confidence interval) indicating good model fit [29]. Values between 0.08 and 0.10 have been suggested as indicating mediocre fit, whereas values above 0.10 indicate poor fit [30]. For SRMR, lower values are also desirable, with values close to 0.08 indicating a good model fit [29].

Apart from investigating goodness-of-fit statistics the magnitude of factor loadings was inspected.

If none of the previously suggested factor solutions would yield satisfactory fit, we decided to proceed by refining the model that would exhibit the most favorable fit in each of our two samples. These model modifications were guided by removing items with factor loadings below 0.4 as well as items that cross-loaded on several factors. The decision to use a cut-off of 0.4 for factor loadings in model modifications was based on common practices, as this is typically considered the lower bound for a substantial relationship between an item and a factor in factor analysis, although there is not any clearly recommended and agreed upon cut-off [31]. Thereafter, modification indices based on residual covariances were inspected for further adjustments. We adopted a conservative approach and only allowed residual covariances when they were theoretically justified, for example when referring to similar meanings or shared structural characteristics, such as negatively formulated items.

### Ethical approval

All studies from which participants were included for the present study had received ethical approval from the Swedish Ethical Review Authority or by the respective Regional Ethics Committees if before 2019 (For the primary care sample; Subsample 1: Dnr: 2019–03786, Subsample 2: Dnr: 845 − 18, Subsample 3: Dnr 2020–03581 and Subsample 4: Dnr 2020–06049 and Dnr 2022-04575-02. For the psychiatric sample; Subsample 1 and 2: LU 864-02 and Subsample 3: Dnr 316/2007. Subsample 4 was part of a comprehensive evaluation of the Swedish mental health services reform in 1995 and initiated by the Swedish Board of Health and Welfare).

## Results

### Primary care sample (Sample 1; *n* = 210)

In subsample 4 item 28 of the Empowerment Scale was missing by design, thus this item includes answers from *n* = 101 in all analyzes for the primary care sample. All other items include answers from *n* = 210 in the analyzes for the primary care sample. The mean ± SD score on the Empowerment Scale for the primary care sample was 2.72 ± 0.31.

### Model 0– one factor based on 28 items

Model 0 showed poor model fit (*χ*^2^_(350)_ = 1074, *p* <.001, CFI = 0.58, TLI = 0.54, RMSEA = 0.10 (90% CI: 0.09 − 0.11), SRMR = 0.11). The pattern of factor loadings was furthermore inconsistent, with many statistically non-significant factor loadings (3, 15, 7, 8, 10, 15, 17, 21, 23) and additionally items with low loadings (< 0.4).

Internal consistency for the whole scale in *sample 1* was high, with coefficient Omega of ω = 0.82 (95% CI: 0.79;0.86) and corresponding Cronbach’s alpha of α = 0.82 (95% CI: 0.79;0.86).

### Model 1– Five-factor solution [11, 14, 15]

Results from this model showed an overall statistically significant goodness-of-fit chi-square statistic (x^2^(337) = 625, *p* <.001). The RMSEA (0.06 with 90% CI 0.06; 0.07) and the SRMR (0.07) indicated acceptable model fit, however the CFI (0.83) and the TLI (0.81) indicated poor model fit.

The pattern of factor loadings was likewise inconsistent with the proposed five-factor structure. Most of the factor loadings in the Power-powerlessness factor were problematic with low loadings, and some were there to statistically insignificant (10, 17, 7). In addition, the factors Optimism and control over the future and Righteous anger also displayed some items with low factor loadings.

The factor Self-Esteem-Self-efficacy displayed high correlation with both Optimism and Control over the future (0.91, *p* <.001) and Power-powerlessness (0.61, *p* <.001), suggesting that this factor was not enough distinct from the other two.

### Model 2– Five-factor model without three items [12]

Results from this model also showed a significant overall goodness-of-fit chi-square statistic (x^2^(262) = 502, *p* = < 0.001), indicating poor model fit to the data. Additional fit statistics showed CFI = 0.85 and TLI = 0.83. RMSEA was 0.07 (90% CI 0.06;0.08) and SRMR was 0.07. Thus, this model did not indicate any substantial difference in model fit statistics compared to model 1. Regarding the pattern of factor loadings, the power-powerlessness factor was still problematic with several very low factor loadings and items 10, 17 and 7, the same items as in model 1, were statistically insignificant. The factor Self-Esteem-Self-efficacy still displayed high correlation with both the Optimism and Control over the future (0.97, *p* <.001) and Power-powerlessness factor (0.61, *p* <.001).

### Model 3. Modified five-factor solution [17]

This model was not able to run in the present sample. The covariance matrix of latent variables was not positive definite.

### Model 4. Three-factor model [18]

The fit indices for this model showed an overall statistically significant goodness-of-fit chi-square statistic (x^2^(270) = 503, *p* = < 0.001), indicating poor model fit to the data. Additional fit indices were for CFI = 0.85 and for TLI = 0.84. RMSEA was 0.06 (90% CI 0.06;0.07) and SRMR was 0.07. All in all, RMSEA and SRMR indicated acceptable fit, however CFI and TLI indicated poor model fit according to suggested limits [29].

Inspecting the factor loadings, the powerlessness-factor was the most problematic factor with two items displaying statistically insignificant loadings (17, 8) and item 21 factor loadings < 0.4. The self-esteem and activism factors were stable, with one item in respective factor displaying low loadings < 0.4 (16 in Self-esteem, 26 in Activism). Item 26 was the item that could cross-load at both Self-esteem and Activism, and according to this sample seemed to fit best in the Self-esteem factor. The factor correlations were low, suggesting distinct factors. The Powerlessness and Activism factors showed a small negative correlation with each other (*r*=-.08, *p* =.42).

Internal consistency for the different subscales according to the three-factor solution in this sample was for Self-Esteem: ω = 0.88 (α = 0.88), for Powerlessness: ω = 0.44 (α = 0.43), and for Activism: ω = 0.84 (α = 0.86).

### Psychiatric sample (Sample 2; *n* = 221)

In a second step, we refit all the models in a sample of patients from psychiatric care in Sweden. The mean ± SD score on the Empowerment Scale for the psychiatry sample was 2.54 ± 0.40.

### Model 0– one factor

Results from the one-factor model in this sample showed an overall statistically significant goodness-of-fit chi-square statistics (x^2^(350) = 1307, *p* = < 0.001), indicating poor model fit to the data. Additional fit indices showed low values, with CFI = 0.66 and TLI = 0.63. RMSEA was 0.11 (90% CI:0.11;0.12) and SRMR was 0.10, both indicating poor model fit. Like in the primary care sample, the matrix of factor loadings in *model 0* displayed several statistically insignificant items (4, 10, 16, 21, 22, 23) and additionally items with low loadings (< 0.4).

Internal consistency for the whole scale in *sample 2* was high, with coefficient Omega of ω = 0.89 and corresponding Cronbach’s alpha of α = 0.88.

### Model 1–3

None of the models 1–3 were able to successfully converge in sample 2. In the case of model 1 and 2, some of the estimated observed variable variances were negative, while for model 3, the estimated covariance matrix was non-positive definite.

### Model 4– three-factor model [18]

Results showed an overall statistically significant goodness-of-fit chi-square statistic (x^2^(270) = 622, *p* <.001). Additional fit indices showed a CFI = 0.87 and TLI = 0.86. RMSEA was 0.08 (90% CI 0.07;0.09) and SRMR was 0.07.

The factor loadings displayed a consistent pattern with those in sample 1 and showed a fair fit with the three-factor solution. Two items within the self-esteem factor (22-, 16) were problematic with statistically insignificant and low loadings. The Powerlessness-factor was as in sample 1 a bit unstable with three items displaying low loadings < 0.4 (21, 17, 8). 

The factor covariances showed a high correlation between the Self-Esteem and Activism factors (*r* =.64, *p* <.001), suggesting these two aspects of empowerment were more interrelated to each other in this sample. Internal consistency for the three factors in this sample was for Self-Esteem: ω = 0.87 (α = 0.86), for Powerlessness: ω = 0.59 (α = 0.57), and for Activism: ω = 0.94 (α = 0.93).

### Refinements of the three-factor solution for respective sample

Based on previous factor solutions, results from our confirmatory factor analyses showed that a three-factor solution with the dimensions *Self-esteem*, *Powerlessness* and *Activism*, which has previously been proposed by Morris and colleagues [18], entailed the least complexity and best model fit in our two samples of participants from primary care and psychiatric care. Although the three-factor solution was judged to be the best of the previous solutions, it also did not provide an acceptable fit in any of our samples and some of the factor loadings were problematic. The internal consistency for the powerlessness factor was additionally very low in both samples, and specifically in the primary care sample. We therefore proceeded by adjusting the model for both samples. First, we removed items with factor loadings below 0.4 as well as factor loadings that cross-loaded on several factors. Second, we inspected the modification indices for further adjustments.

### Adjusted model for the primary care sample (sample 1)

The adjusted model resulted in a three-factor solution with 20 items. Items 16 (self-esteem), 21, 17, 8 (powerlessness), 3 and 26 (activism) were removed because they displayed factor loadings below < 0.4. Because we removed item 16 in the “trimmed model”, we also removed the residual correlation between item 16 and 22 that was proposed in Morris and colleagues’ model [18]. We also removed the cross loading for item 26, which in our model only loaded high enough in the self-esteem factor. Instead, in our model a residual correlation was allowed between items 11 and 28, which both refer to collectively changing things in society.

The adjusted model for the primary care sample showed model fit indices of (x^2^(166) = 307, *p* <.001), CFI = 0.90, TLI = 0.89, RMSEA = 0.06 (90% CI 0.05;0.08), SRMR = 0.07, all in all indicating acceptable model fit. Internal consistency for the three factors in this model was for Self-Esteem: ω = 0.87 (α = 0.87), for Powerlessness: ω = 0.56 (α = 0.56), and for Activism: ω = 0.86 (α = 0.89).

### Adjusted model for the psychiatric care sample (sample 2)

The adjusted model for the psychiatric sample resulted in a three-factor solution with 20 items. We removed items 16, 22 (self-esteem) and 21, 17, 8 (powerlessness) based on low factor loadings (< 0.4). Item 26 was additionally removed from the Self-esteem factor based on its cross-loading in two factors but was kept in the Activism factor where it had its highest loading (as opposed to in the primary care sample, where it was kept in the Self-esteem factor). Like in the primary care sample (sample 1), item 16 was removed in our adjusted model and thus also the residual correlation between items 16 and 22 that was proposed in Morris and colleagues’ model [18]. Based on the modification indices, residual correlations were allowed between items item 11 and 28 (which both refer to changing things in society through cooperation) and between items 20 and 25 (which both refer to individuals having the right to decide for themselves).

The model fit indices for the adjusted model in the psychiatric sample was (x^2^(165) = 498, *p* <.001), CFI = 0.87, TLI = 0.85, RMSEA = 0.10 (90% CI 0.09;0.11) and SRMR = 0.08. All in all, the fit indices for CFI and TLI were poor, whereas RMSEA and SRMR together were more acceptable. Internal consistency for the three factors in this model was for Self-Esteem: ω = 0.90 (α = 0.90), for Powerlessness: ω = 0.63 (α = 0.60), and for Activism: ω = 0.93 (α = 0.92). Table 2 shows the factor loadings of the adjusted models for both samples respectively.

**Table 2:** Adjusted factor solutions for different Samples^1^

Factor	Item	Description^2^	Primary Care (Sample 1)		Psychiatric Care (Sample 2)
Self-esteem			Factor loading		
	1	I can pretty much determine what will happen in my life (rev)	0.46	0.49	
	5	I have a positive attitude about myself (rev)	0.69	0.77	
	6	I am usually confident about the decision I make (rev)	0.50	0.61	
	9	I see myself as a capable person (rev)	0.78	0.83	
	12	I am often able to overcome barriers (rev)	0.66	0.61	
	13	I am generally optimistic about the future (rev)	0.65	0.70	
	14	When I make plans, I am almost certain to make them work (rev)	0.47	0.42	
	18	I am able to do things as well as most other people (rev)	0.62	0.66	
	19	I generally accomplish what I set out to do (rev)	0.61	0.59	
	22	I feel powerless most of the time	0.57		
	24	I feel I am a person of worth, at least on an equal basis with others (rev)	0.76	0.78	
	26	I feel I have a number of good qualities (rev)	0.73		
Powerlessness					
	4	Getting angry about something never helps	0.63	0.76	
	7	People have no right to get angry just because they don’t like something	0.52	0.51	
	8	Most of the misfortunes in my life were due to bad luck			
	10	Making waves never gets you anywhere	0.50	0.48	
	17	Experts are in the best position to decide what people should do or learn			
	21	You can’t fight city hall			
Activism					
	3	People have more power if they join together as a group (rev)		0.72	
	11	People working together can have an effect on their community (rev)	0.74	0.90	
	20	People should try to live their lives the way they want to (rev)	0.75	0.80	
	25	People have a right to make their own decisions, even if they are bad ones (rev)	0.62	0.69	
	26	I feel I have a number of good qualities		0.52	
	27	Very often a problem can be solved by taking action (rev)	0.72	0.89	
	28	Working with others in my community can help to change things for the better (rev)	0.73	0.88	
^1^Adjusted models based on the factor solution by Morris et al., 2014					
^2^rev= reverse the scorings such that a 4 = 1, 3 = 2, 2 = 3 and 1 = 4					

### One adjusted model for the two samples

Since there were just slight differences between the adjusted models for the two samples respectively, our last step involved removing the items that differed between the samples (items 22 and 26 in the self-esteem subscale and items 3 and 26 in the Activism subscale) and refitting this model in the respective samples. This was motivated by a practical aim in arriving at a solution of the scale that could be used within health care contexts in general, regardless of primary care or psychiatric care level. In this final model, the residual correlation between items 11 and 28 (changing things in society) was retained because this was the residual correlation included in both the adjusted models for sample 1 and sample 2, while the residual correlation between items 20 and 25 was removed because this residual correlation only was found in the adjusted model for the psychiatric sample (Sample 2).

The model fit indices for this model were for the primary care sample: (x^2^(131) = 240, *p* <.001), CFI = 0.91, TLI = 0.90, RMSEA = 0.06 (90% CI 0.05;0.08) and SRMR = 0.07, indicating overall acceptable model fit. Internal consistency for the three factors in the primary care sample was Self-Esteem: ω = 0.87 (α = 0.87), for Powerlessness: ω = 0.56 (α = 0.56), and for Activism: ω = 0.86 (α = 0.89). For the psychiatric care sample, the model fit indices were: (x^2^(131) = 379, *p* <.001), CFI = 0.88, TLI = 0.86, RMSEA = 0.09 (90% CI 0.08;0.10) and SRMR = 0.07. Thus, fit indices for CFI and TLI were still poor for the psychiatric sample, but RMSEA and SRMR were acceptable and lower than our first adjusted model for the psychiatric sample, indicating somewhat better model fit. Internal consistency in the psychiatric sample was for Self-Esteem: ω = 0.89 (α = 0.89), for Powerlessness: ω = 0.63 (α = 0.60), and for Activism: ω = 0.93 (α = 0.92).

As a last step, we tested this final model on our whole sample, combining the primary care and psychiatric care sample (*n* = 431). The model fit indices for this model were (x^2^(131) = 432, *p* <.001), CFI = 0.91, TLI = 0.90, RMSEA = 0.07 (90% CI 0.07;0.08) and SRMR = 0.06, overall indicating acceptable model fit. Internal consistency for the three factors based on this model and in our combined sample was for Self-Esteem: ω = 0.88 (α = 0.88), for Powerlessness: ω = 0.59 (α = 0.58), and for Activism: ω = 0.92 (α = 0.92).

Table 3 shows the factor loadings for the final model fitted in both samples respectively, as well as when fitted in these samples combined.

**Table 3:** Factor loadings for one adjusted model for both samples**

Factor	Item	Description	Primary Care (Sample 1)	Psychiatric Care (Sample 2)	Combined sample (1 + 2)	
Self-esteem			Factor loading			
	1	I can pretty much determine what will happen in my life (rev)	0.46	0.51	0.49	
	5	I have a positive attitude about myself (rev)	0.69	0.77	0.70	
	6	I am usually confident about the decision I make (rev)	0.52	0.63	0.58	
	9	I see myself as a capable person (rev)	0.77	0.81	0.81	
	12	I am often able to overcome barriers (rev)	0.66	0.62	0.66	
	13	I am generally optimistic about the future (rev)	0.66	0.71	0.69	
	14	When I make plans, I am almost certain to make them work (rev)	0.49	0.44	0.48	
	18	I am able to do things as well as most other people (rev)	0.64	0.67	0.67	
	19	I generally accomplish what I set out to do (rev)	0.63	0.61	0.63	
	24	I feel I am a person of worth, at least on an equal basis with others (rev)	0.74	0.76	0.76	
Powerlessness						
	4	Getting angry about something never helps	0.63	0.76	0.70	
	7	People have no right to get angry just because they don’t like something	0.52	0.51	0.52	
	10	Making waves never gets you anywhere	0.50	0.47	0.48	
Activism						
	11	People working together can have an effect on their community (rev)	0.71	0.89	0.85	
	20	People should try to live their lives the way they want to (rev)	0.76	0.81	0.81	
	25	People have a right to make their own decisions, even if they are bad ones (rev)	0.64	0.70	0.70	
	27	Very often a problem can be solved by taking action (rev)	0.72	0.90	0.86	
	28	Working with others in my community can help to change things for the better (rev)	0.71	0.85	0.82	
*Adjusted models based on the factor solution by Morris et al., 2014						

## Discussion

With this study we aimed to evaluate the Swedish adaptation of the Empowerment Scale – Making Decisions. The purpose of the study was to assess its structural validity and reliability in measuring patient empowerment in health care settings. Concretely, our aim was to make a psychometric assessment of the scale. The scale has been evaluated in psychiatric populations of people with serious mental illness, but to our knowledge no study has evaluated the psychometric properties in a clinical primary care population. Psychiatric and primary care patients may have diverse needs and experiences in healthcare, which can impact their self-perceived empowerment. In this study, we evaluated the scale’s structural validity and dimensionality as well as its internal consistency on two samples of patients receiving mental health care in either primary care or psychiatric care. We used confirmatory factor analysis (CFA) to replicate four factor solutions proposed in previous research to identify the best fit for the two samples. In addition, we tested a one-factor solution to assess the unidimensionality of the scale.

The findings from this study showed that none of the previously proposed factor solutions yielded satisfactory fit in either our primary care or psychiatric sample. Among the four proposed solutions, the three factor-solution with the constructs *Self-Esteem*, *Powerlessness* and *Activism*, previously found in [18], entailed the less complexity and best model fit in both our two samples (p*rimary care sample*: (x^2^(270) = 503, *p* = < 0.001),CFI = 0.85, TLI = 0.84, RMSEA = 0.06 (90% CI 0.06;0.07), SRMR = 0.07; *psychiatric care sample*: (x^2^(270) = 622, *p* <.001), CFI = 0.87, TLI = 0.86, RMSEA = 0.08 (90% CI 0.07;0.09), SRMR = 0.07). However, because these models still did not yield satisfactory fit, we continued to explore the factor structure in an exploratory manner in our two samples. This involved refining the models by examining modification indices and eliminating items with low factor loadings (< 0.4) or double loadings. This iterative process allowed us to find two refined and decent models for the primary care and psychiatric care sample, respectively. The two models were overall similar between the two samples, except for three items that differed (items 22, 3 and 26). Thus, in a last step we removed the items that differed between the samples to arrive at one solution appropriate for use in health care settings in general. This was motivated by the fact that the two adjusted models for respective samples displayed only slight differences, and a pragmatic motive in arriving at a solution of the scale that could be used within health care contexts in general. Despite differences between primary care and psychiatric care settings, such as a focus on holistic health, preventive measures, and addressing a wide range of medical concerns within primary care, and more emphasis on symptom management, medication, and psychotherapeutic interventions within psychiatric care, there are also commonalities shared between the samples. While the specific healthcare goals may differ, both groups aspire to improve their health, well-being, and overall quality of life through their respective care experiences. In both settings, there is a recognition of the importance of patient autonomy and the need to involve patients in making informed choices about their healthcare. The fundamental objective in both settings is to provide patient-centered care that respects individual autonomy and preferences.

We refit this last generic model in the two samples respectively, and in a final step also on the two samples combined (*n* = 431). Through this, we explored both the fusion and the separation of these two populations, acknowledging the aspects in which they are distinct as well as those in which they exhibit similarities. The refined solution included 18 items based on the subscales *Self-Esteem*, *Powerlessness* and *Activism* and displayed an overall acceptable model fit (although less favorable model fit in the psychiatric care sample) both when fitting the model in the two samples respectively (*primary care sample*: x^2^(131) = 240, *p* <.001, CFI = 0.91, TLI = 0.90, RMSEA = 0.06 (90% CI 0.05;0.08), SRMR = 0.07; *psychiatric care sample*: x^2^(131) = 379, *p* <.001, CFI = 0.88, TLI = 0.86, RMSEA = 0.09 (90% CI 0.08;0.10), SRMR = 0.07) and when combining the samples and treating them as one population (x^2^(131) = 432, *p* <.001), CFI = 0.91, TLI = 0.90, RMSEA = 0.07 (90% CI 0.07;0.08), SRMR = 0.06). Internal consistency for the subscales were high for both Self-Esteem (*primary care*: ω = 0.87 (α = 0.87), *psychiatric care*: ω = 0.89 (α = 0.89), *combined*: ω = 0.88 (α = 0.88)) and Activism (*primary care*: ω = 0.86 (α = 0.89), *psychiatric care*: ω = 0.93 (α = 0.92), *combined*: ω = 0.92 (α = 0.92)), however less satisfactory for the Powerlessness-subscale (*primary care*: ω = 0.56 (α = 0.56), *psychiatric care*: ω = 0.63 (α = 0.60), *combined*: ω = 0.59 (α = 0.58)).

Several considerations arise from the findings of this study. Firstly, the results from the one-factor solution (model 0) in both samples offer limited support for the scale’s unidimensionality. This has both practical and theoretical implications. The scale is intended to serve as a unitary measurement, providing a total empowerment score. However, based on the findings of this study as well as previous research, there are doubts about whether the scale truly represents a single construct, and it may better measure various related aspects. If the scale cannot be considered to measure a uniform concept, it also becomes problematic to calculate an overall score of empowerment for practical use. The scarce support for the scales unidimensionality is however not surprising given previous research on empowerment as a multifaceted concept, entailing several aspects, and dimensions of both individual and psychological, political, contextual and social aspects [3, 4, 5, 10]. Existing measurement tools for empowerment cover a range of different domains and related concepts, which reflect that there is no uniform definition of the concept to date [5, 9, 10]. Previous research on The Empowerment Scale – Making decisions, as well as the results in this study, have shown high internal reliabillity for the whole scale. However, reliability measures do not account for scale dimensionality, rather it is a measure of how well items correlate with each other. Items may correlate because they measure the same thing, which is ideally what these measures are used for signaling. However, a high internal reliabillity could also be because the items measure different things that are correlated with each other. With regards to the Empowerment Scale, items assessing self-esteem may also corrleate with items measuring societal acitvism. Additionally, reliabillity measures like cronbach’s alpha are affected by the number of scale items, with more items resulting in higher alpha.

Further, because empowerment is such an elusive concept, it is not surprising that in previous evaluations of the scale it has been difficult to find a satisfactory and uniform factor solution. The fact that we could not confirm any of the previous suggested solutions in our two samples may be related to different contexts where the scale has been tested, different populations, different countries, different backgrounds and theoretical domains of the researchers, or different ways of statistically analyzing the data (for example principal component analysis/CFA, orthogonal/oblique rotation). Because the perception of empowerment may be highly influenced by the context and the population being considered, some researchers have emphasized that empowerment instruments need to be specifically developed and adapted to the context in which they should be used [4]. The results of this study could be interpreted as aligning with this view and supporting the fact that we should not expect to find a generic one-size-fits-all measurement tool of empowerment. The Empowerment Scale – Making decisions was developed in the late 1990s [11]. It is possible that patients in today’s healthcare have a different view of empowerment, and to a greater extent expect to be involved and participate in their own care. Further, cultural differences may significantly influence how empowerment is understood and experienced, particularly in healthcare settings. The original Empowerment Scale – Making Decisions was developed in the U.S, where much of health care is privatized. In Sweden, the healthcare system is publicly funded. These factors may influence how patients perceive their role in decision-making processes and their interactions with healthcare providers. For example, in Sweden the concept of patient empowerment might be shaped by the country’s strong emphasis on egalitarianism and individual rights and the emphasis may be on collaborative decision-making within a universal healthcare framework. Additionally, the original instrument entails inquiries about sense of empowerment on a macro level, and an ambition to investigate how patients with severe mental illness perceive their empowerment in relation to their integration in society. Other items are likely to become more relevant when assessing empowerment in a health care relationship, such as questions about shared decision making and patients’ feelings of influence within their own health care. In fact, previous research on the scale found that the model fit improved with second-order constructs referring to a psychological state of empowerment on one hand, and societal and political power on the other hand. Hansson and Björkman [14] found a five-factor solution with two superordinate factors, one revolving around “self-esteem and activism” and one directed towards “community and power”, suggesting that some items relate more to a psychological state of empowerment with links to constructs such as self-efficacy and self-esteem, while others relate more to community action and political power. Likewise, Corrigan and colleagues [16] found that a six-factor solution yielded two subordinate factors: a self-orientation to empowerment and a community-orientation to empowerment. Ideally, future research could develop an assessment tool which is aimed more precisely to measure patient empowerment within a health care relationship. A more specific instrument for health care context would also be more successful when looking to capture change in patients’ sense of empowerment as a result of health care interventions, as change in a specific care context does not necessarily equal change in empowerment at a general or macro level.

Consequently, the study’s findings make it clear that there is still an open question how patient empowerment, as a result of different contexts or interventions, can be measured specifically in a health care setting. In this study we arrived at a modified version of the Empowerment Scale – Making Decisions that displayed acceptable model fit and factor loadings. However, it is doubtful if such an instrument provides a comprehensive picture of empowerment as a concept. There are numerous theories among researchers about what empowerment is, or should be, based on a certain perspective or overarching theory. However, it appears that it cannot be demonstrated that this construct exists in the minds of individuals, that is, that it resonates with how people themselves make sense of their experiences. Furthermore, beyond the practical measurement challenges associated with factors like self-esteem and self-efficacy within the construct of empowerment, there is a compelling argument to consider excluding them from the conceptual framework of empowerment entirely. This suggestion stems from a growing body of empirical findings, emphasizing the need to acknowledge and act upon the consequences of this empirical reality. For example, in our final refined three-factor model derived from our empirical results, the instrument puts emphasis on subscale self-esteem, with more than half of all items measuring this facet. Given that there are already numerous well-validated scales that measure self-esteem, such as the Rosenberg Self-Esteem Scale [32], these may offer a more comprehensive assessment of this construct. Additionally, self-efficacy can also be measured with other instruments (for example: the general self-efficacy scale [33] and the new general self-efficacy scale [34]). However, as previously discussed, based on the literature on empowerment we perceive this concept to be broader than just confidence in one’s own abilities. Empowerment is context-dependent, shaped and influenced by the environment. Thus, future research could explore ways to measure empowerment beyond these constructs.

Additionally, given that it is doubtful whether empowerment could even be understood as a unitary concept, future research needs to reconsider the value of empowerment as a concept, especially when concerning a health care context, and consider if empowerment may be better captured with several different concepts and measurement tools. As an example, in another study, we found that an internet-delivered CBT intervention for primary care patients with anxiety disorders that was tailored based on participants’ self-selection influenced perceived control over treatment [19]. The control questions were specifically developed for the study, and an instrument with related questions could be a way to examine patient empowerment in relation to primary care treatments. Examples of questions were “How much impact do you feel you have had on the overall treatment plan?”, “How much impact do you feel you have had on the content of the treatment?” and “Would you have liked to have more influence over the structure of the treatment?” [19]. Another existing and promising measurement instrument with similar questions is the “Patient Enablement Instrument” [35]. Questions in this instrument includes “As a result of your visit to the doctor today, do you feel that you are able to cope with your illness” and “As a result of your visit to the doctor today, do you feel that you are confident about your health” [35]. These and comparable questions could be a way forward for measuring patient empowerment within a health care setting. Another approach could involve starting with basic research, by interviewing patients about their perception of factors influencing patient agency and patient influence in health care contexts.

### Methodological considerations and limitations

Measurement instruments ideally need to be psychometrically evaluated in the context where it is supposed to be used. To our knowledge the Empowerment scale – Making decisions has not been evaluated on a primary care patient population before, and this is a strength of the present study. The empirical results from this study gave a similar picture about the scale’s dimensionality in both the primary care and psychiatric care sample and an adjusted solution based on three factors which remained relatively clearer to pervious research was put forward. Internal consistency was high for the subscales Self-esteem and Activism, both when fitting the model in our two samples respectively and when fitting it to our combined sample. However, the subscale for Powerlessness showed questionable internal consistency both in the respective samples: primary care: ω = 0.56 (α = 0.56), psychiatric care: ω = 0.63 (α = 0.60) and combined: ω = 0.59 (α = 0.58), suggesting that this subscale is still problematic. This aligns with previous studies that likewise have obtained questionable internal consistency for this subscale [13, 14, 15]. The results of this study thus suggest an adjusted solution of the scale; however, this needs to be further replicated in independent samples.

This study also has limitations. Our two samples were relatively small, and more participants would have been preferable when replicating the factor structures proposed in previous research. It was methodologically not possible to test some of these factor solutions in our psychiatric sample, probably due to a limited sample size relative to the complexity of these models. Another consideration is that the two primary care and psychiatric care samples comprised participants recruited for different research studies, and it is always dubious to conclude that they represent two uniform populations. The primary care sample had a gender bias towards women, which is partly because one study only entailed women (subsample 4, which was comprised of pregnant women with depression) and could also be explained by more women presenting with common mental disorders, as often seen in primary care [36]. Since empowerment is seen as a contextual concept which may be affected by power imbalances, social norms and so forth, a reasonable hypothesis is that empowerment may differ between men and women. Additionally, most of the subsamples for the primary care sample, except for subsample 3 (*n* = 35), had been recruited for research studies on internet-delivered treatment programs. Internet-delivered treatment programs are largely based on self-help, with limited therapist support [37]. It could be hypothesized that patients enlisted for this type of treatment may present with higher self-efficacy, which could contribute to higher empowerment scores, compared to primary care patients seeking other forms of treatment modalities. Furthermore, the psychiatric study participants were enlisted from 2007 to 2014, in contrast to the primary care sample, which included individuals recruited within the past five years. Given the possibility that patients’ relationship to and experience of empowerment may have evolved over the last 15 to 20 years, this could be another factor influencing the outcomes.

Finally, in our modified models presented for the primary care and psychiatric care samples respectively, and for the whole sample combined, we made modifications based on low factor loadings and modification indices. The decisions made based on the modification indices are inevitably also subjected to interpretations. In addition to the residual covariance that we allowed in both our samples between item 28 and 11, two additional residual covariances were suggested for the primary care sample that seemed reasonable. Our notion is that items 14 and 6 could have been allowed a residual correlation based on its temporal form (both refer to the future), and items 19 and 12 could be allowed to have a residual correlation based on that both refer to a goal-directed behavior. These residual covariances could have further improved the model fit. However, we decided on a conservative line and chose to have as few residual covariances as possible.

## Conclusions

In summary, the findings from this study suggest an improved and shortened adaptation of the Empowerment Scale - Making decisions that could potentially be used within health care settings to measure empowerment. The refined solution of the scale entailed a three-factor solution with the subscales Self-Esteem, Activism and Powerlessness. However, internal consistency for the subscale Powerlessness was questionable. The proposed factor solution remains relatively clear to the proposed solutions in previous research in our samples of primary care and psychiatric care patients, but further research is needed to explore how empowerment is conceptualized and could be measured in health care settings for patients with mental health problems. Overall, the results from this study reinforce the difficulties in measuring empowerment given the complexity of this concept which consists of several different and related aspects. We suggest future research to reconsider if the concept of empowerment may better be measured with several different measurement instruments targeting different constructs as well as to investigate how factors influencing patient empowerment specifically within a health care context can be measured.

## Electronic supplementary material

Below is the link to the electronic supplementary material.

Supplementary Material 1

Supplementary Material 2

Supplementary Material 3

## Data Availability

The datasets analysed during the current study are available from the corresponding author on reasonable request.

## Abbreviations

CFA: Confirmatory factor analysis
CFI: Comparative fit index
ICBT: Internet delivered cognitive behavioral therapy
RCT: Randomized controlled trial
RMSEA: Root mean square error of approximation
SRMR: Standardized root mean square of the residuals
TLI: Tucker-Lewis index
