# Supplementary material for: Empowerment in primary care and psychiatric settings: a psychometric evaluation of the Swedish version of the empowerment scale
Source: BMC Psychol. 2025 Aug 13;13:909. doi: 10.1186/s40359-025-03123-y (PMC12345097; doi:10.1186/s40359-025-03123-y)
Supplement: Supplementary file 2 — Supplementary Material 2 [file 40359_2025_3123_MOESM2_ESM.docx]

| **Appendix II.** | |
| --- | --- |
| *Final adjusted model of The Empowerment Scale for the two samples* | |
|  |  |
| Item | Description* |
|  |  |
| 1 | I can pretty much determine what will happen in my life (rev) |
| 4 | Getting angry about something never helps |
| 5 | I have a positive attitude about myself (rev) |
| 6 | I am usually confident about the decision I make (rev) |
| 7 | People have no right to get angry just because they don't like something |
| 9 | I see myself as a capable person (rev) |
| 10 | Making waves never gets you anywhere |
| 11 | People working together can have an effect on their community (rev) |
| 12 | I am often able to overcome barriers (rev) |
| 13 | I am generally optimistic about the future (rev) |
| 14 | When I make plans, I am almost certain to make them work (rev) |
| 18 | I am able to do things as well as most other people (rev) |
| 19 | I generally accomplish what I set out to do (rev) |
| 20 | People should try to live their lives the way they want to (rev) |
| 24 | I feel I am a person of worth, at least on an equal basis with others (rev) |
| 25 | People have a right to make their own decisions, even if they are bad ones (rev) |
| 27 | Very often a problem can be solved by taking action (rev) |
| 28 | Working with others in my community can help to change things for the better (rev) |
|  |  |
| * rev = reverse the scorings such that a 4=1, 3=2, 2=3 and 1=4 | |
|  |  |
