# Supplementary material for: Empowerment in primary care and psychiatric settings: a psychometric evaluation of the Swedish version of the empowerment scale
Source: BMC Psychol. 2025 Aug 13;13:909. doi: 10.1186/s40359-025-03123-y (PMC12345097; doi:10.1186/s40359-025-03123-y)

**Appendix III**

**Primary care sample (sample 1)**

*Model 0*


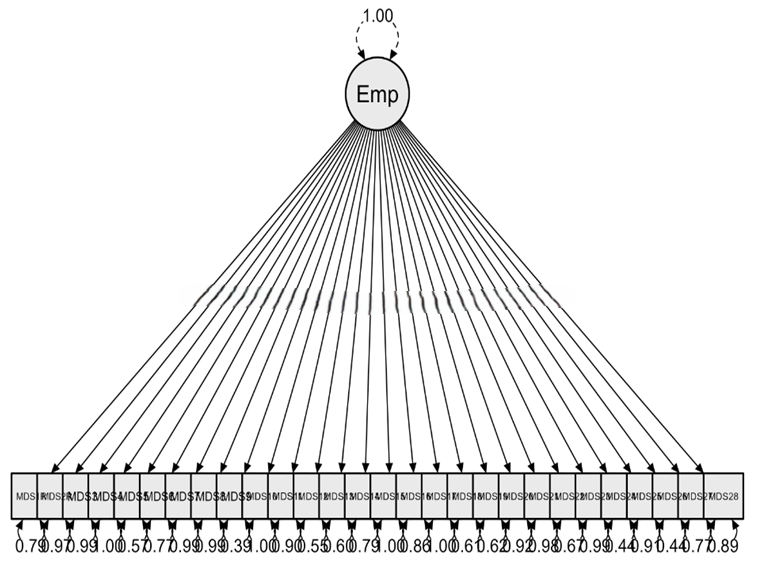


*Model 1 – Rogers et al., 1997*


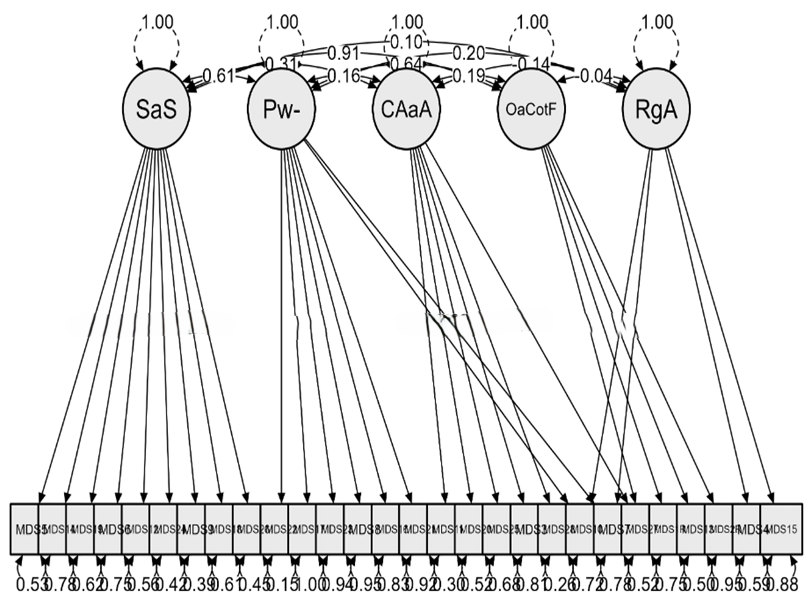


*Model 2 – Rogers et al., 2010*


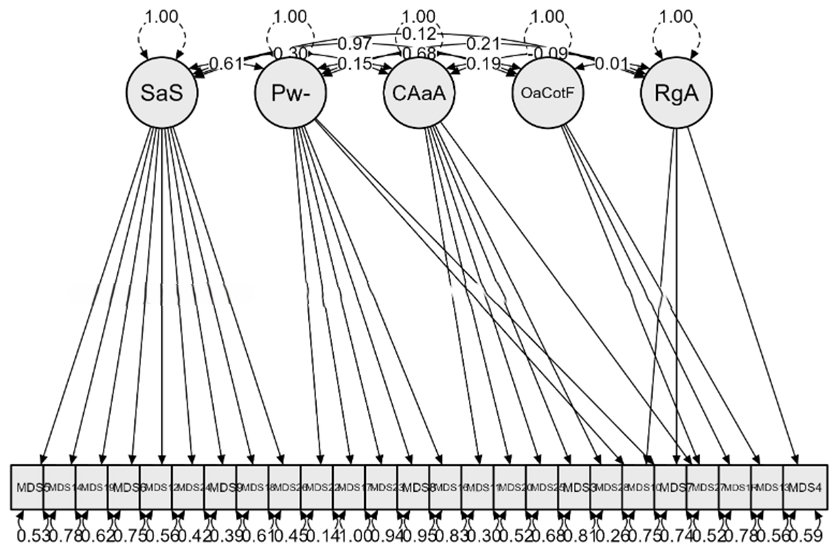


*Model 4 – Morris et al., 2014*


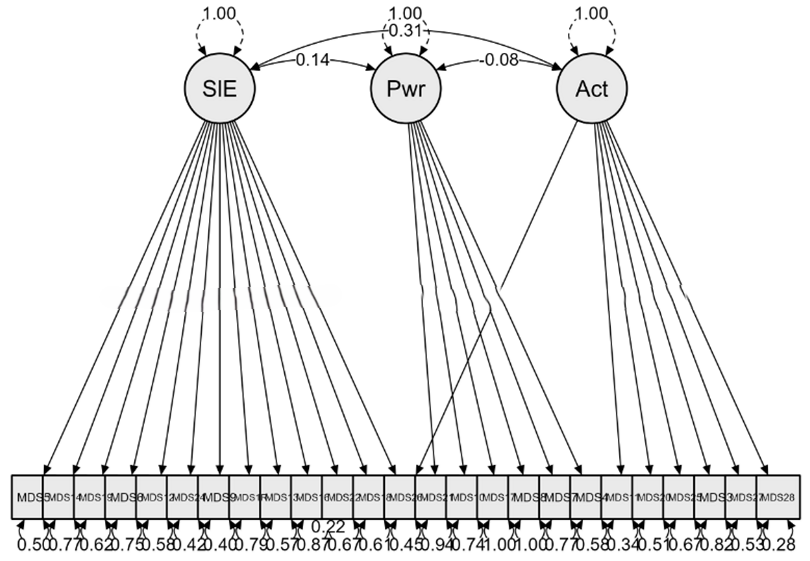


**Psychiatric care sample (sample 2)**

*Model 0*


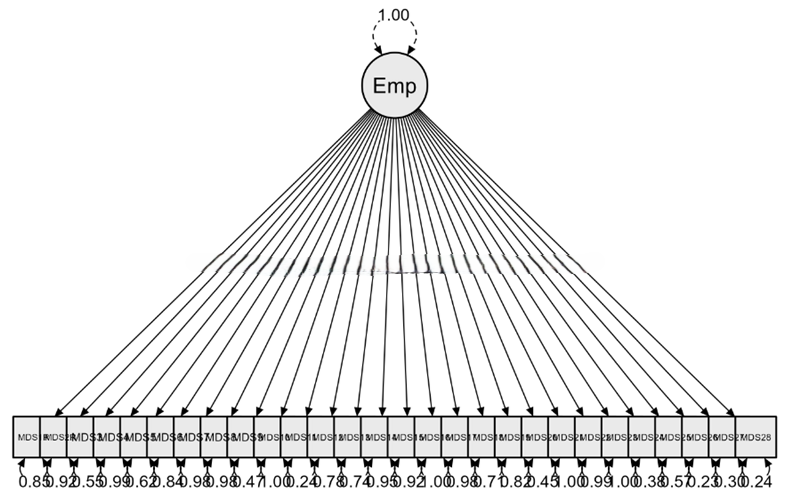


*Model 4 - Morris et al., 2014*


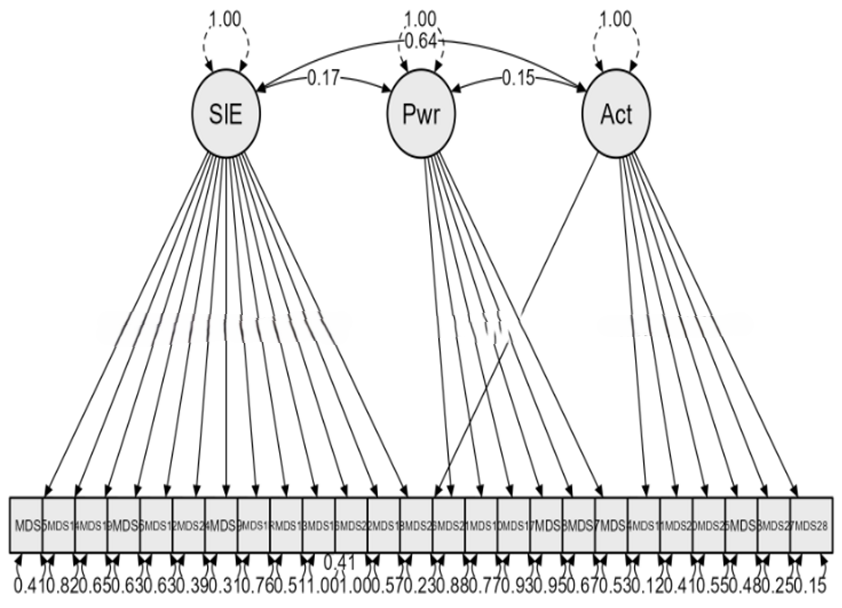


**Adjusted model of the three-factor solution proposed by Morris et al., 2014**

*Primary care sample (sample 1)*


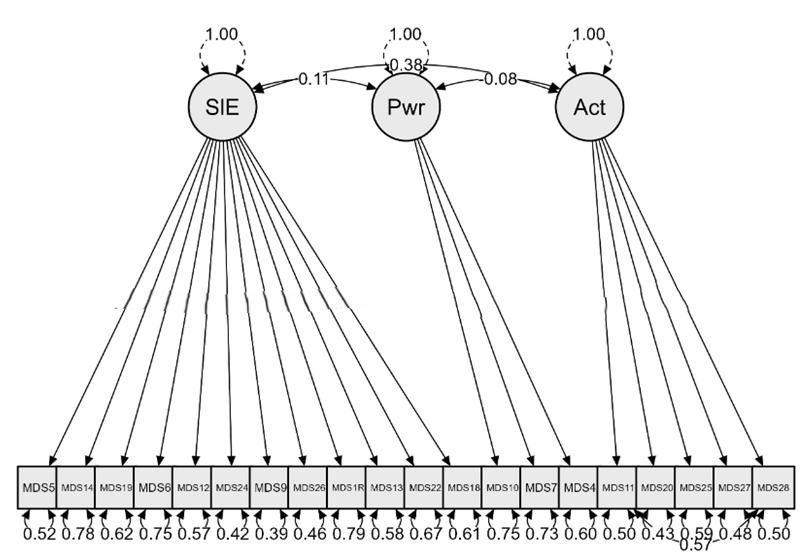


*Psychiatric care sample (sample 2)*


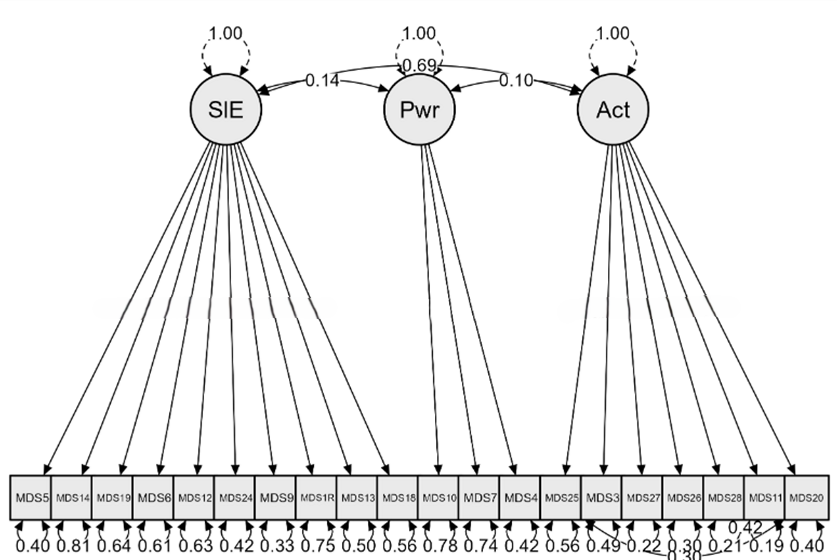


**One adjusted model for the two samples**

*Primary care sample (sample 1)*


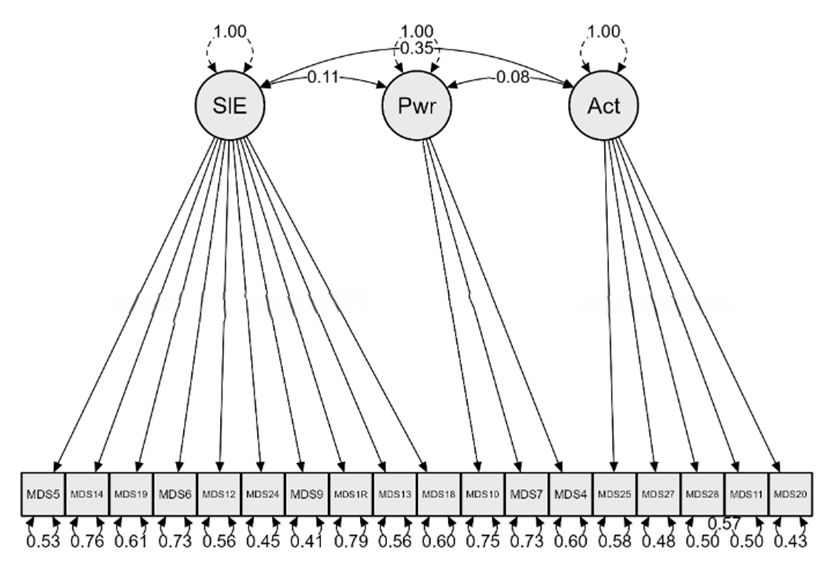


*Psychiatric care sample (sample 2)*


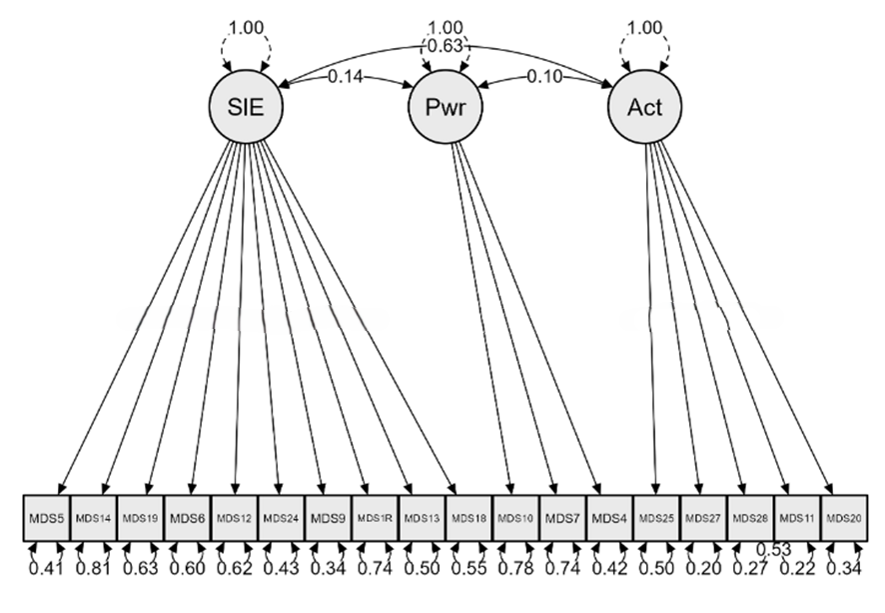


*Combined sample (sample 1 and sample 2 together, n=431)*


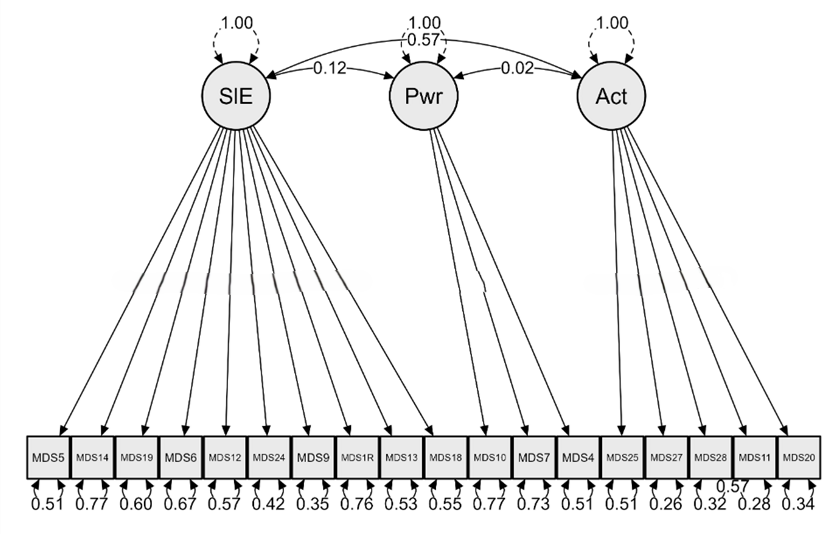

Supplement: Supplementary file 3 — Supplementary Material 3 [file 40359_2025_3123_MOESM3_ESM.docx]
